# Excellent Energy Storage Performance in Epoxy Resin Dielectric Polymer Films by a Facile Hot−Pressing Method

**DOI:** 10.3390/polym15102315

**Published:** 2023-05-15

**Authors:** Zhe Pan, Minmin Mao, Bin Zhang, Zhongyu Li, Kaixin Song, Hai-Feng Li, Zhu Mao, Dawei Wang

**Affiliations:** 1 College of Electronics Information, Hangzhou Dianzi University, Hangzhou 310018, China; 2 Shenzhen Institute of Advanced Electronic Materials, Shenzhen Institute of Advanced Technology, Chinese Academy of Sciences, Shenzhen 518055, China; 3 School of Physics and Information Technology, Shaanxi Normal University, Xi’an 710119, China; 4 Institute of Applied Physics and Materials Engineering, University of Macau, Avenida da Universidade, Taipa, Macao SAR 999078, China; 5 School of Instrumentation Science and Engineering, Harbin Institute of Technology, Harbin 150080, China

**Keywords:** epoxy resin, polymer film, hot pressing, capacitor

## Abstract

Epoxy resin (EP), as a kind of dielectric polymer, exhibits the advantages of low-curing shrinkage, high-insulating properties, and good thermal/chemical stability, which is widely used in electronic and electrical industry. However, the complicated preparation process of EP has limited their practical applications for energy storage. In this manuscript, bisphenol F epoxy resin (EPF) was successfully fabricated into polymer films with a thickness of 10~15 μm by a facile hot−pressing method. It was found that the curing degree of EPF was significantly affected by changing the ratio of EP monomer/curing agent, which led to the improvement in breakdown strength and energy storage performance. In particular, a high discharged energy density (*U_d_*) of 6.5 J·cm^−3^ and efficiency (*η*) of 86% under an electric field of 600 MV·m^−1^ were obtained for the EPF film with an EP monomer/curing agent ratio of 1:1.5 by hot pressing at 130 °C, which indicates that the hot−pressing method could be facilely employed to produce high−quality EP films with excellent energy storage performance for pulse power capacitors.

## 1. Introduction

Dielectric capacitors are widely used in electronic and electrical industry because of their merits of high−power density, short charge–discharge rate, long service life and high operating voltage [1,2,3,4]. As basic electronic components, dielectric capacitors are commonly found in electric power systems, hybrid vehicles, oil drilling, and sophisticated weapon systems [5,6,7]. With the continuous development of modern electronics and power systems, there is an urgent need to continuously enhance the performance of energy storage materials, such as discharged energy density (*U_d_*) and efficiency (*η*) [8,9,10]. It is well known that *U_d_* can be calculated by Equations (1) and (2) for nonlinear and linear materials, respectively [11,12,13,14], while *η* can be calculated by Equation (3).

(1) $$U_{d}=\int_{D_{r}}^{D_{\max}}EdD$$

(2) $$U_{d}=\frac{1}{2}DE=\frac{1}{2}\varepsilon_{0}\varepsilon_{r}E^{2}$$

(3) $$\eta =\frac{U_{d}}{U_{d}+U_{loss}}\times 100\%$$

where the *D*_max_, *D_r_*, *E*, *D* are the maximum electric displacement, the remanent electric displacement, the applied electric field, and the electric displacement of dielectric material, respectively [15,16]; *ε*_0_, *ε_r_* are the dielectric constant of the free space and the relative dielectric constant, respectively [17,18]; *U_loss_* is the energy loss. From Equation (2), it is well known that the *U_d_* of dielectric materials is mainly determined by breakdown strength (*E_b_*) and dielectric constant (*ε_r_*) [19,20,21]. Therefore, in order to improve *U_d_*, numerous studies have been conducted to increase *ε_r_* and *E_b_* [22].

There are many polymers that are widely prepared as dielectric capacitors due to some of their properties, such as BOPP film which has a low dielectric constant (~2.2 @ 1 kHz), high breakdown field strength, and fine hysteresis line, and energy storage density of 5 J·cm^−3^ (@ 25 °C) [23]. The polar material PVDF has a high dielectric constant (~10 @ 1 kHz) and high polarization, so it is often used as energy storage material, but because of its weak electric field resistance, the pure PVDF film energy storage density is usually below 10 J·cm^−3^ (@ 25 °C). These polymers are the relatively mainstream raw materials currently used in ambient temperature energy storage capacitors. These are considered thermoplastics that can be purchased directly from pharmaceutical companies. However, these materials need to be dissolved with some solvents such as DMF, NMP, and DMAc. These solvents are usually accompanied by volatilization during the experimental process and therefore have a certain impact on the environment, and are relatively expensive and not suitable for mass research or industrial production [24]. In summary, we tried to develop the use of thermosetting plastics for energy storage performance studies. However, due to its electrical properties and the selection of a suitable curing agent for a cross−linking reaction, epoxy resin (EP) can be used for energy storage applications [25]. Therefore, this article focuses on the application of energy storage by changing the preparation scheme, including the ratio of raw materials and hot−pressing conditions, under the condition that the epoxy resin monomer and curing agent are prepared into bisphenol F epoxy resin (EPF). 

However, EP can usually only be prepared as bulky blocks due to their complicated processing, which greatly limits *E_b_*. Consequently, it is of great significance to explore a new preparation method to reduce the thickness of EP. Polymer composites composed of EP and Ag nanoparticle−modified Al_2_O_3_ microspheres (Al_2_O_3_-AgNPs) were reported by Ren et al., where the *E_b_* was decreased from ~203 MV·m^−1^ for pure EP to ~78 MV·m^−1^ for the 70 wt% Al_2_O_3_/EP composite [26]. Hexagonal boron nitride (h−BN) and graphite oxide (GO) were electrostatically assembled by Huang et al. to prepare a new h−BN−RGO hybrid as a reinforcement, which was added to EP to obtain a composite with high thermal conductivity, high *ε_r_*, and low *tanδ*. However, the *E_b_* of the composite was low, which was found to decrease from 41 MV·m^−1^ to 32.31 MV·m^−1^ with the increase in hybrid content [27]. A new A−B−A−type composite was fabricated by Zhao et al., in which *ε_r_*, *E_b_*, and *U_d_* were reported to be greatly improved. The A layer was a composite prepared by the oriented carbon nanotube bundles (ACB) reinforced EP (ACB/EP), and the B layer was a composite obtained by adding dopamine−coated barium titanate fibers to EP (PDA@BTnf/EP). Among all the materials, the *E_b_* of A−10B−A was the highest, reaching 6.09 MV·m^−1^, and the maximum *U_d_* of A−2B−A could reach 0.1148 J·cm^−3^ [28]. After Tomer first hydrothermally modified the barium titanate nanoparticles (BaTiO_3_, BT), the modified BT and organically modified montmorillonite were added to the EP in different ratios and then centrifugally mixed, followed by the addition of curing agent to complete the curing reaction by hot pressing. Eventually, it was found that the energy storage performance of the composite films was relatively stable under different ratios of inorganic particles, and the maximum *U_d_* was around 2.5 J·cm^−3^ under an electric field of 320 MV·m^−1^ [29]. Different concentrations of imidazole with (2−ethyl−4−methylimidazole, 2E4MZ) or without (1−eth−ylimidazole, 1EZ) pyrrole−type nitrogen were selected and homogeneously mixed with Bisphenol−A epoxy resin E51 by Luo et al. The *U_d_* of EP−2E4MZ was 4.4 J·cm^−3^ with an *η* of less than 60% @ 500 MV·m^−1^ [30].

Studies have shown that the *E_b_* of dielectric materials is negatively related to their thickness [31,32]. However, the EP thickness reported in these studies exceeds that in this work, which leads to low *E_b_* and deteriorated energy storage performance. In this work, bisphenol F epoxy resin (EPF) is selected as a demonstration to study the effect of processing parameters on the preparation, microstructure, and energy storage properties of EP thin films by a facile hot−pressing method. EPF has the characteristics of low viscosity, high−electrical insulation, corrosion resistance, good adhesion, and excellent mechanical properties of cured products [33,34,35]. However, the epoxy resin itself will only cure under certain conditions, and a curing agent mixed with cross−linking reaction, in order to cure into a certain size of the sample and have the required performance, and the epoxy polymer performance is generally affected by the nature of the curing agent [36,37,38,39,40,41]. Compared with other types of curing agent, anhydride curing agent has low volatility, causes little skin irritation, and has a low toxicity, and when mixed with EP, the viscosity is relatively low; it is a convenient experimental operation, the shrinkage rate is small, and no by−products are formed in the experimental process. In addition, there are several anhydride−type curing agents for their own state of liquid, and the experimental mixture only needs to be fully stirred at a certain temperature to complete the experiment. Therefore, this experiment included the anhydride curing agent selection, and finally selected the weather resistance and leakage resistance traces with excellent performance for the curing agent. After the selection of suitable EP and curing agent in the early stage, through a series of experiments to adjust the ratio of raw materials and curing the hot−pressing conditions, the following experimental conclusions were finally reached. It was well revealed that both the *ε_r_* and *E_b_* of EPF could be effectively improved by adjusting the curing agents and conditions. Finally, a record high of *E_b_*~600 MV·m^−1^ was obtained for EPF thin film with an EPF monomer/curing agent ratio of 1:1.5 and curing temperature of 130 °C, leading to an ultrahigh *U_d_* of 6.5 J·cm^−3^ and *η* of 86%.

## 2. Materials and Methods

### 2.1. Preparation of EPF Film

**Figure 1:**
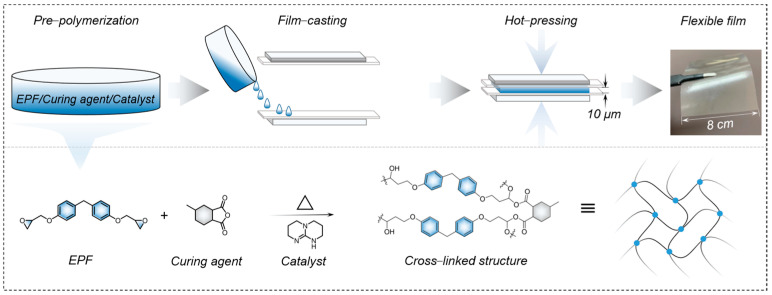
Preparation diagram of EPF film.

The raw materials used for the experiment were bisphenol F epoxy resin (EPF, Nippon Steel & Sumikin Chemicals Co., Ltd., Tokyo, Japan), 5−Methylhexahydroisobenzofuran−1,3−dion (C_9_H_12_O_3_, ≥98%, Bide Pharmatech Co., Ltd., Shanghai, China), and 2,3,4,6,7,8−Hexahydro−1H−pyrimido [1,2−a]pyrimidine (C_7_H_13_N_3_, ≥97%, Bide Pharmatech Co., Ltd., Shanghai, China). The preparation of EPF film is schematically shown in Figure 1. An amount of 1 mol of EPF monomer has two EP functional groups, and 1 mol of this anhydride curing agent has an anhydride functional group. Through the formula m = M * n, the mass of the EP is fixed, and the mass of the curing agent corresponding to the ratio of different functional groups is calculated. The ratio of 1 EP functional group to a functional anhydride group (a = 0.5, 0.85, 1.2, 1.4, 1.6, 1.8) is expressed as 1:a. The calculated mass ratio 1:α is the experimentally actual weighed mass of EP and curing agent, where α = 0.5, 0.9, 1.3, 1.5, 1.7, and 1.9, abbreviated as α EPF. Initially, EPF and C_9_H_12_O_3_ were weighed and added to the aluminum box in a calculated ratio to obtain the EPF slurry, and then the mixed slurry with a total mass of 1 wt% and the amine catalyst C_7_H_13_N_3_ was quickly added to the EPF slurry. The EPF slurry was heated and stirred at 130 °C until the catalyst was completely dissolved, and then the temperature slowly increased to 150 °C for a total of about 10 min to complete the pre−curing step. At the same time, the hot platen covered with PET film was preheated at the subsequent EPF curing temperature until the temperature stabilized and the EPF completed the pre−curing step. The EPF slurry was then poured into a square mold with a thickness of 10 μm, which was placed on the hot platen covered with PET film, and hot pressed at 110 °C, 130 °C, and 150 °C, and at 5 MPa, 10 MPa, and 15 MPa, respectively, for 2 h. Finally, the prepared EPF films were peeled off after the finished cured films were cooled to room temperature.

### 2.2. Characterization

The morphology of the EPF film was observed by a scanning electron microscope (SEM, Apreo 2 S, Thermo Fisher Scientific, Waltham, MA, USA). The EPF film was submerged in liquid nitrogen for 30 min and torn off to obtain the cross section for SEM images. X−ray diffraction (XRD, Bruker D8 Advance, Billerica, MA, USA) was performed at 40 kV and 40 mA to detect the phase structure. Fourier-transform infrared spectroscopy (FTIR, Invenio R, Bruker, Billerica, MA, USA) was performed in attenuated total reflection mode. For electrical measurement, gold electrode with a diameter of 2 mm was sputtered on both sides of samples. A precision impedance analyzer (Agilent E4980A, Santa Clara, CA, USA) was used to measure the dielectric properties. E_b_ was determined by a DC withstanding voltage tester (eec7474, Taiwan EXTECH Electronics, Taiwan, China). Ferroelectric hysteresis loops were tested at a frequency of 100 Hz using a ferroelectric test system (PolyK Technologies, State College, PA, USA). Mechanical properties were tested with the Dynamic Thermomechanical Analyzer—Full Mode (DMA850, TA Instruments, New Castle, DE, USA). The glass transition temperature (Tg) of the epoxy resin film was determined using a differential scanning calorimeter (with light curing device) apparatus at a heating rate of 10°C/min (DSC2500, TA Instruments, New Castle, DE, USA).

## 3. Results

### 3.1. Different Ratios of Curing Agent to EPF

**Figure 2:**
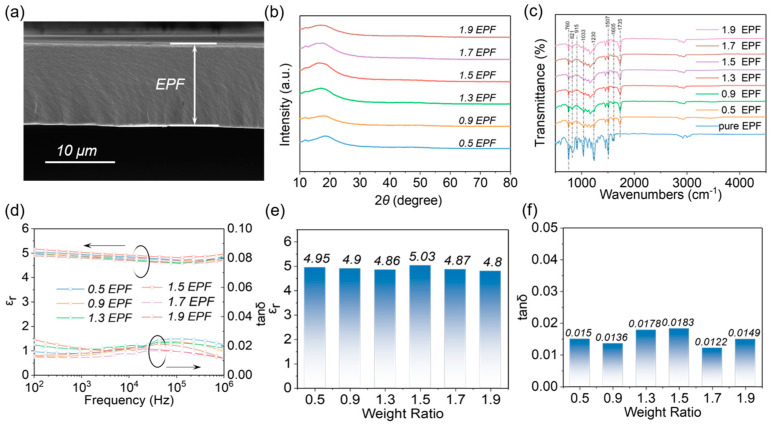
(**a**) SEM image of 1.5 EPF; (**b**) XRD patterns; (**c**) FTIR spectra; (**d**) variation in *ε_r_* and *tanδ* of EPF films with different values of α; numerical analysis of *ε_r_* (**e**) and *tanδ* (**f**) of α EPF at 1 kHz (α is the mass ratio of EP and curing agent, 1: α).

**Figure A1:**
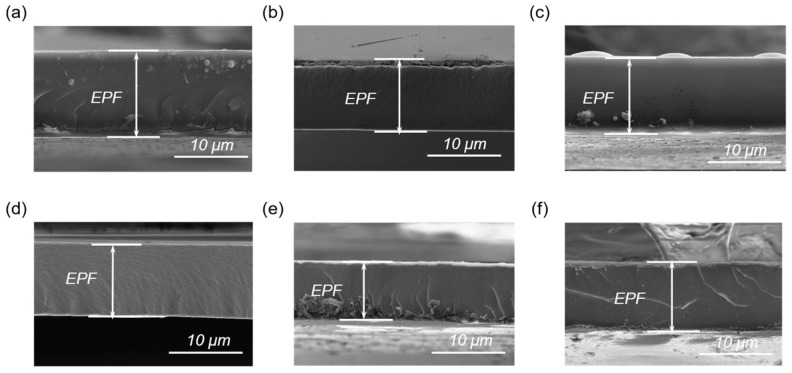
SEM images of 0.5 EPF (**a**); 0.9 EPF (**b**); 1.3 EPF (**c**); 1.5 EPF (**d**); 1.7 EPF (**e**); 1.9 EPF (**f**).

**Figure A2:**
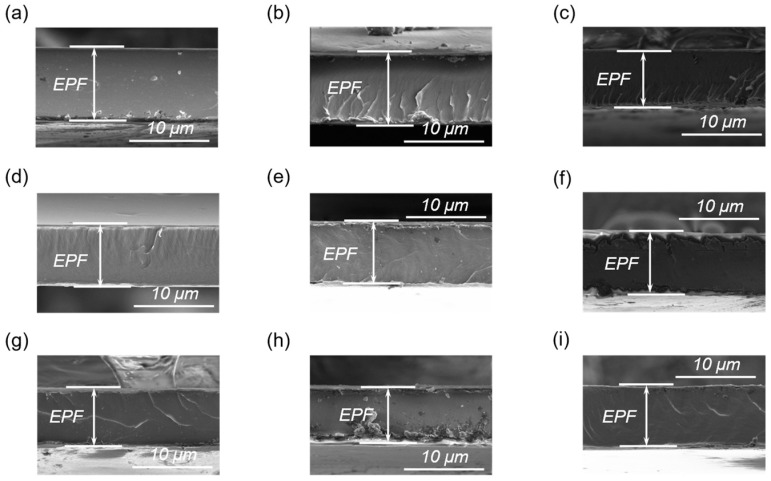
SEM images of 110 °C, 5 MPa (**a**); 110 °C, 10 MPa (**b**); 110 °C, 15 MPa (**c**); 130 °C, 5 MPa (**d**); 130 °C, 10 MPa (**e**); 130 °C, 15 MPa (**f**); 150 °C, 5 MPa (**g**); 150 °C, 10 MPa (**h**); 150 °C, 15 MPa (**i**).

**Figure A3:**
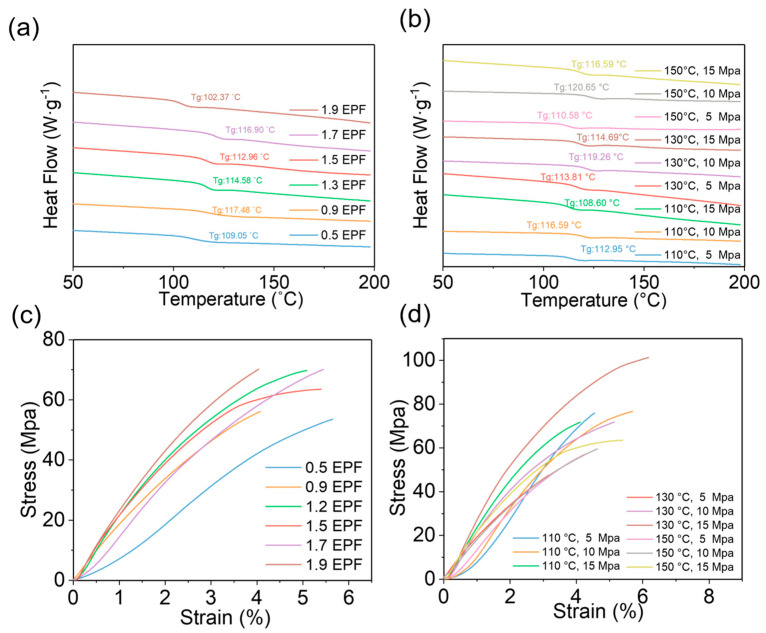
The DSC (**a**) and DMA (**c**) of α EPF; the DSC (**b**) and DMA (**d**) for samples with different hot−pressing temperatures and pressures.

Figure 2a shows the SEM images for the cross sections of EPF films, which indicate that the films are dense and homogeneous with a thickness of about 10 μm. Figure A1 and Figure A2 show the SEM images for other scales and different conditions of EP films, which are of different thicknesses, with 0.9 EPF, 1.3 EPF, and 1.5 EPF having smoother cross sections, but in general, essentially showing homogeneous cross sections without holes, and some of the films do not have completely flat surfaces because they are all pure EP films. The non−crystalline nature of EPF films has a dispersion peak at ~18° [42], which is confirmed by the XRD pattern [38], and the intensity of this dispersion peak tends to increase and then decrease with the increase in the components, with the maximum intensity at 1.5 EPF. The FTIR spectra of EPF films are given in Figure 2c. The stretching of CH_2_ and CH_3_ groups can be seen below 3000 cm^−1^ [43,44,45]; 1507 cm^−1^ and 1605 cm^−1^ correspond to the absorption of the C=C group in the benzene ring [46]; 1230 cm^−1^ is caused by the stretching vibration of the in−plane deformation of C-O of the phenolic group. The medium and broad bands out at 1033 cm^−1^ are caused by the stretching vibration of the aliphatic C−O functional group [47]. Meanwhile, the strong peak at 810 cm^−1^ is assigned to be the C−H bending vibration of the aromatic group. The characteristic peak of −NH stretching can be seen at 2800–3000 cm^−1^ and the characteristic peak of the benzene ring in EP is at 760 cm^−1^, which is present in all EP samples. At 915 cm^−1^, the characteristic peak of the epoxy group is shown, which is very obvious in pure EP, and disappears with the FTIR of α EPF when the curing agent is added and the epoxy group is cross linked with the anhydride group. There is no characteristic peak at 1735 cm^−1^ in the FTIR of pure EP, but a characteristic peak appears after the addition of curing agent, which is the C=O bond of the anhydride group, and the characteristic peak gradually increases with the increase in the mass ratio of the curing agent. In Figure A3a, the Tg of α EPF was measured by DSC equipment, and it can be seen that the Tg of all the overall samples was around 110 °C. It was found that when α was 0.9–1.7, the Tg of the samples was relatively concentrated at 117.48 °C, 114.58 °C, 112.96 °C, and 116.90 °C, respectively, while when α was extremely large or small, the Tg of the samples was relatively small at 109.05 °C (α = 0.5) and 102.37 °C (α = 1.9). This may be due to the inappropriate mass ratio and imbalance in the ratio of raw materials, which makes the epoxy group too much (α = 0.5) or the anhydride group too much (α = 1.9) to affect its curing degree, thus further affecting its Tg. In Figure A3c, the mechanical properties of the samples obtained by DMA test could be seen. Three main indicators were referenced from the figure, the strain and stress at the mechanical break of the film were defined as the elongation at break (σ) and the ultimate tensile strength (λ) of the sample, respectively, and the Young’s modulus (Y) of the sample was obtained by fitting the slope of the curve [48]. From the figure, we can see that λ exceeds 50 MPa for all samples, up to 70 MPa, and σ is above 4% for all of them, and the highest is only 5.6%, which is different from thermoplastic polymers with σ above 10%, probably because the EP has a highly cross−linked molecular structure with less σ. Y, with the exception of 0.5 EPF and 1.9 EPF, is concentrated in 1400–1500 MPa, which is the same trend as that measured by DSC above, probably also because the ratio is too large or too small, resulting in the completion of the film−curing reaction of the hot pressing not being as successful as other ratios. From the above DSC and DMA results, it can be seen that the mechanical and thermal properties of the samples will be affected to a certain extent when the raw material ratios are changed, but the difference in performance is not particularly large within a certain ratio range. The frequency dependence of *ε_r_* and dielectric loss (*tanδ*) for EPF films with different values of α can be seen in Figure 2d. It shows that the *ε_r_* and *tanδ* of the samples vary slightly with the increase in frequency from 100 Hz to 1 MHz. Meanwhile, at the frequency of 1 kHz, as α increases from 0.5 to 1.9, the *ε_r_* and *tanδ* generally change slightly, as shown in Figure 2e,f. The maximum *ε_r_* and *tanδ* of 5.03 and 0.0183 were obtained by the 1.5 EPF sample, in agreement with the reported value [27,48]. From the above analysis of the epoxy resin film mechanical properties, thermal properties, and this part of the film’s dielectric properties, it can be concluded that the relationship between the curing agent and the EP monomer ratio will affect the mechanical and thermal properties of the epoxy resin, while affecting its structure, which further affects its dielectric properties.

**Figure 3:**
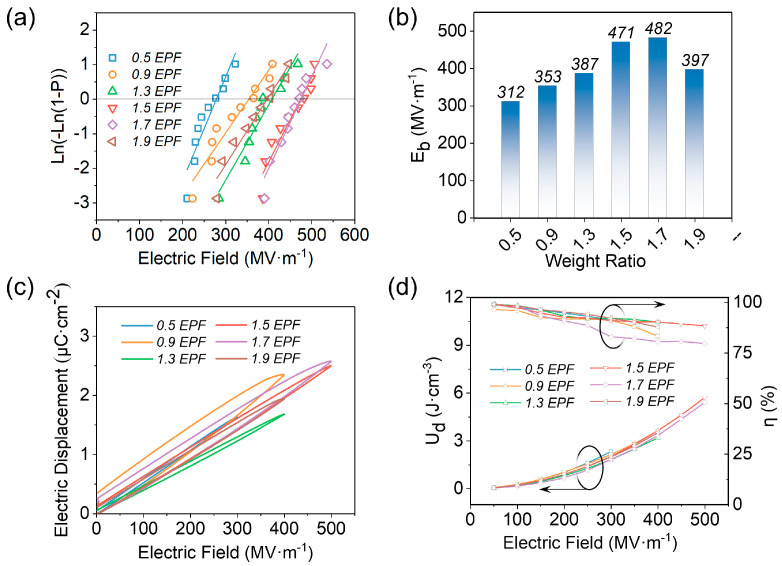
(**a**) Weibull distribution; (**b**) *E_b_*; (**c**) hysteresis loops; (**d**) *U_d_* and *η* of EPF films with different values of α (α is the mass ratio of EP and curing agent, 1: α).

From Equations (1) and (2), it is known that the *U_d_* of a material has a positive correlation with its *E_b_*. The *E_b_* of EPF can be obtained from the Weibull distribution, as shown in Equation (4):

(4) $$P(E)=1-\;\exp[-(\frac{E}{E_{0}}{)}^{\beta }]$$

where *P(E)* denotes the cumulative breakdown probability, *E* denotes the breakdown intensity of the sample when tested (*E_b_*), *E_0_* is the Weibull breakdown field strength, and *β* denotes the shape parameter of the sample breakdown data dispersion [48,49,50]. *E_b_* of the EPF films with different α values are plotted in Figure 3a. It shows that with the increase in α value, *E_b_* increases at first and then decreases. The *E_b_* of 0.5 EPF, 0.9 EPF, 1.3 EPF, 1.5 EPF, 1.7 EPF, and 1.9 EPF ratios were 312 MV·m^−1^, 353 MV·m^−1^, 387 MV·m^−1^, 471 MV·m^−1^, 482 MV·m^−1^, and 397 MV·m^−1^, with 1.7 EPF having the highest *E_b_* relative to 0.5 EPF increased by 55.5% (Figure 3b), which indicates that the *E_b_* of EPF films can be effectively improved by changing the ratio of raw materials. The hysteresis loops of EPF films at their maximum electric fields are shown in Figure 3c. It shows that the hysteresis loop of 1.5 EPF is slimmer than that of 1.7 EPF at the same electric field, suggesting a larger *η*. The corresponding energy storage performance of *U_d_* and *η* is calculated and plotted in Figure 3d. The *η* is more than 80% for all samples, and in particular, the *η* for 1.5 EPF is greater than those of other compositions due to the slim hysteresis loop. Ultimately, the maximum *U_d_* of 5.72 J·cm^−3^ is achieved by 1.5 EPF with an *η* of 88.4% at 500 MV·m^−1^, which is a 145.5% increase in the *U_d_* of 2.33 J·cm^−3^ for 0.5 EPF. This is because 1.5 EPF has the highest *ε_r_* and one of the highest *E_b_* compared to the rest of the ratios, and according to the calculation formula, its *U_d_* is the highest and the hysteresis line is the thinnest, which is equivalent to the maximum *η*.

### 3.2. Different Hot−Pressing Conditions

**Figure 4:**
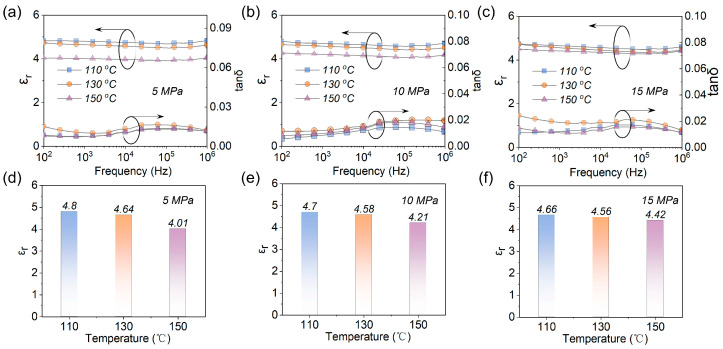
*ε_r_* and *tanδ* for the EPF samples with different hot−pressing temperatures at (**a**,**d**) 5 MPa, (**b**,**e**) 10 MPa, and (**c**,**f**) 15 MPa.

Based on the experimental data and conclusions in the previous section, 1.5 EPF was selected to further investigate the thermal compression conditions on the energy storage properties of EP films. In the preparation process of EPF films, there are two key parameters for hot pressing: temperature and pressure, which are both studied in this section. In Figure A2, it can be seen that changing the pressure and temperature changes the morphology of the film more than adjusting the raw material ratio, and the cross section of the film is smoother and basically free of other impurities at 5 MPa. As can be seen from Figure A3b,d, when the ratio of EP monomer to curing agent was determined to be 1.5 EPF, Tg, σ, λ, and Y changed significantly when only the temperature and pressure of hot pressing were adjusted, indicating that the hot−pressing conditions, as with the raw material ratio, would affect the mechanical and thermal properties of EP. Figure 4a–c show the frequency dependence of *ε_r_* and *tanδ* of the EPF samples with different hot−pressing temperatures under the pressure of 5 MPa, 10 MPa, and 15 MPa, respectively. The *ε_r_* of EPF samples with different hot−pressing temperatures shows a slight decrease with the increase in frequency from 100 Hz to 1 MHz. Furthermore, at the frequency of 1 kHz, with the increase in hot−pressing temperature, the *ε_r_* of EPF samples is also shown to decrease at different pressures (Figure 4d–f), among which the difference between *ε_r_* of the 110 °C and 130 °C hot−pressed EPF samples is not significant.

**Figure 5:**
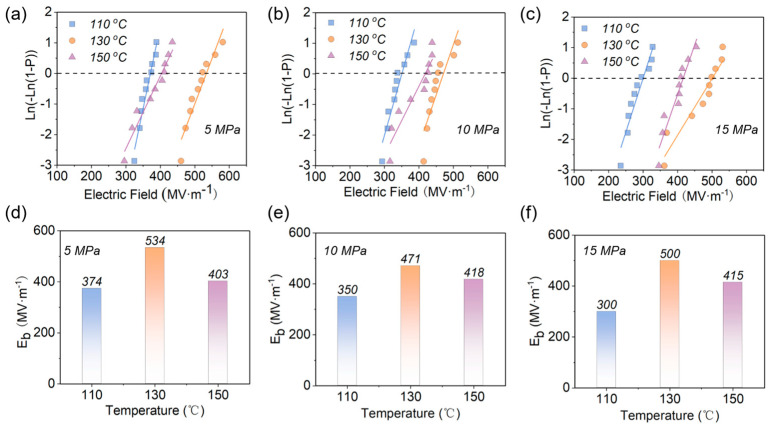
Weibull distribution and *E_b_* at (**a**,**d**) 5 MPa, (**b**,**e**) 10 MPa, and (**c**,**f**) 15 MPa for samples with different hot−pressing temperatures.

**Figure 6:**
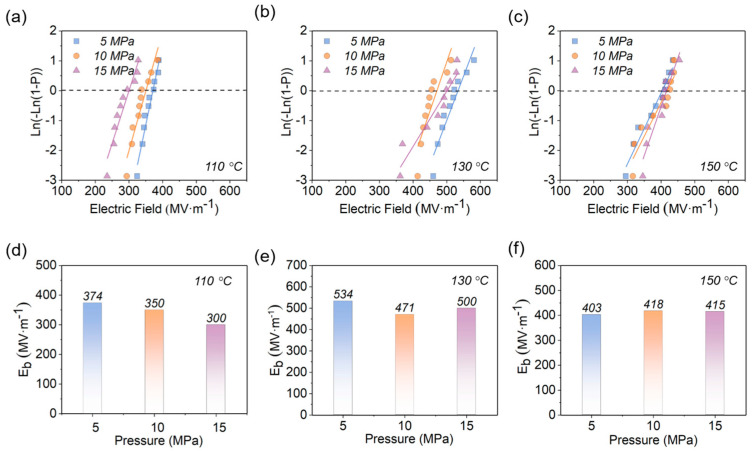
Weibull distribution and *E_b_* at (**a**,**d**) 110 °C, (**b**,**e**) 130 °C, and (**c**,**f**) 150 °C for samples with different hot−pressing pressures.

The Weibull distribution of the EPF samples with different hot−pressing temperatures and pressures are plotted in Figure 5 and Figure 6, which clearly show that the hot−pressing temperature has a large effect on *E_b_*. Taking the pressure of 5 MPa as an example (Figure 5d), the *E_b_* of the samples hot−pressed at 110 °C, 130 °C, and 150 °C are 374 MV·m^−1^, 534 MV·m^−1^, and 403 MV·m^−1^, respectively, in which the highest value is ~143% of the lowest one. However, it can be seen from Figure 5 and Figure 6 that the hot−pressing pressure does not have significant influence on *E_b_.* For instance, the *E_b_* of the samples is slightly decreased from 534 MV·m^−1^ for 5 MPa to 500 MV·m^−1^ for 15 MPa at the hot−pressing temperature of 130 °C (Figure 6e). Consequently, it can be concluded that the *E_b_* of EPF films is dominated by the hot−pressure temperature, and the pressure is only a secondary influence. In particular, it should be noticed that the maximum *E_b_* of 534 MV·m^−1^ was achieved when the hot−pressing pressure was 5 MPa and the hot−pressing temperature was 130 °C.

**Figure 7:**
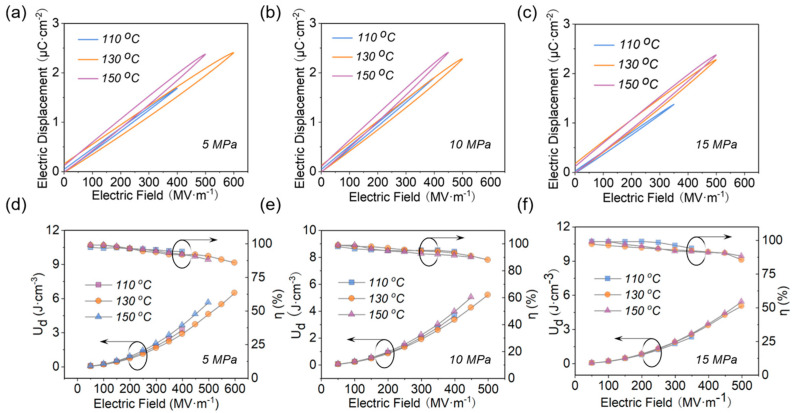
Hysteresis loops (**a**,**b**,**c**) and *U_d_* and *η* (**d**,**e**,**f**) for samples with different hot−pressing temperatures and pressures.

The hysteresis loops of EPF films with different hot−pressing temperatures and pressures at the maximum electric field are shown in Figure 7a–c. It shows that *D_max_* is increased with the increase in hot−pressing temperature at different pressures. The corresponding energy storage properties of the EPF films obtained under different preparation conditions are shown in Figure 7d–f. It can be seen that the *η* of studied samples all exceeds 85%, which benefits from the slim hysteresis loops (Figure 7a–c). With the increase in electric field, *U_d_* is significantly increased and η is gradually decreased. In particular, the samples prepared at a hot−pressing temperature of 130 °C have a more stable *η*, which can reach a higher value at high field strengths. When the pressure is 5 MPa, the maximum *U_d_* of 6.50 J·cm^−3^ with a high *η* of 86% can be finally obtained.

## 4. Conclusions

In this work, EPF films with a thickness of ~10 μm were successfully prepared by a facile hot−pressing method. The effect of compositions and preparation conditions on the microstructure, phase structure, dielectric properties, ferroelectric properties, and energy storage properties were investigated in detail. Firstly, it was found that adjusting the ratio of EPF monomer and anhydride curing agent significantly affected the dielectric, ferroelectric, and energy storage properties. When the mass ratio of the EPF monomer and curing agent was 1:1.5, the *U_d_* of the film could reach 5.71 J·cm^−3^, and *η* was as high as 88.4%. Furthermore, after fixing the mass ratio, a higher *E_b_* was achieved at a hot−pressing temperature of 130 °C relative to other temperatures. Finally, it was determined that the excellent energy storage performance of *U_d_* = 6.5 J·cm^−3^ and *η* = 86 % could be obtained under the electric field of 600 MV·m^−1^ at 130 °C and 5 MPa. The results indicate that the energy storage performance of EPF film can be effectively optimized by adjusting the raw materials and preparation conditions, employing a facile hot−pressing method.

## Data Availability

Not applicable.
